# Preventing dementia? Critical perspectives on a paradigm of preparing for old age

**DOI:** 10.1177/1471301221989600

**Published:** 2021-01-30

**Authors:** James R. Fletcher

**Affiliations:** 4616 King’s College London

Over recent years, dementia has slipped ever earlier into the lifecourse. As popular aetiological hypotheses have faltered, various stakeholders have suggested that underlying pathologies begin decades before cognitive impairment. This has amplified interest in preventive strategies, targeting midlife interventions at future dementias. *Preventing dementia* offers timely critical insight into this ‘new dementia’ – a predictable and preventable midlife disease process. All academics in dementia studies will benefit from this book, and while background knowledge is required to get the most out of it, there is also considerable fodder for scholars across the medical social sciences, that is the reconceptualisation of ageing and the limits of responsibilisation.

The book is split into three sections: dealing with (1) social practises of prevention, (2) prediction and early detection and (3) prevention’s basic conceptual premises. Introducing the collection, Leibing and Schicktanz suggest that the recent popularity of dementia prevention demarcates a new dementia, softening traditional distinctions between dementia and the lifecourse, while foregrounding holistic imaginings of neurocognitive entanglements with biologies, environments, socialities and polities. The authors caution, however, that the potential of this approach depends on how new dementia is envisaged, exhibited and enacted. This cautiousness sets the tone for the book.

The first section opens with Keuck charting historic conceptualisations of Alzheimer’s disease (AD) and their implications for promises of prevention. She argues that new dementia contains and sustains suspect notions of pathology, symptomology and nosology that have been replaced with various working assumptions for a century. Today’s AD is a working title that informs our contemporary efforts but will likely change again. Working titles facilitate the accrual of resources, the generation of institutional prowess and justifications for related practises. This argument is an important counterbalance to more traditional histories of AD.

In chapter two, Leibing considers the post-2010 emergence of scientific consensus on prevention as a departure from neuroreductionism, driven by the increased prominence of cardiovascular pathologies in mainstream aetiological thinking. The chapter draws on interviews with Brazilian health professionals to unpack intersecting state and personal responsibilities for prevention, highlighting morally charged ascriptions of irresponsibility to disadvantaged populations. Crucially, Leibing notes the potential for prevention to challenge representations of dementia as democratic by highlighting unequal landscapes of risk.

Schicktanz’s chapter considers the German turn to prevention, associated ethical uncertainties and overlapping discourses. Germany has relied on established approaches to genetic prediction as akin to biomarker prediction, but prevention complicates matters with questions of individual, professional and state responsibility for present determinants of future (ill-)health. Media discourses cast new dementia as simplistic risk factors and prevention strategies: preached by professionals and practised by individuals. However, while embracing the positive health messaging and conduciveness to research funding, professional healthcare discourse emphasises multimodality and caution regarding new dementia. This distinction reveals the situatedness of prevention.

Leanza considers critiques of neoliberal governmentality that have long been applied to post-industrial preventive health strategies. He argues that new dementia requires a reassessment of ‘neoliberalism’ as critique. Preventive actions are nebulous (e.g. get a good education and avoid getting depressed) and uninspiring, so that prevention is only practicable in public forums. Moreover, increased individual responsibility does not inherently entail decreased state responsibility. The preventive self and preventive collective are codependent because one’s capacity to prevent relies on epidemiological projects, communication campaigns, government legislation and cultural values.

Blasimme pursues a gerontological approach to dementia vis-à-vis ageing and continuum, exploring how new dementia redraws normal/pathological boundaries in relation to geriatric interest in frailty and geroprotectors. Frailty is increasingly associated with dementia, bolstering the notions of longitudinal ecological and biological aetiology that characterise new dementia. This ‘geriatric logic’ underpins the pursuit of geroprotectors, promising pharmacological prevention. This promise reconfigures ageing, pathology and dementia, so that we must disrupt our own ageing ever earlier in the lifecourse.

Part two begins with Moreira on mild cognitive impairment (MCI). Initially, a precursor to AD that would facilitate future research, MCI has become a condition in its own right. Historically, it held together neurological, clinical, therapeutic and market interests, but it has drifted away from promissory biotech to more pragmatic concerns of managing cognitive decline, with diagnosis leading to lifestyle and planning advice. Interviews with clinicians reveal how patients drove MCI through increased diagnosis-seeking, and how MCI can satisfy anxious older people seeking solutions to perceived decline. This counters traditional depictions of MCI as technoscience-led, and partly explains its continued clinical popularity.

Katz, Peters and Ballantyne consider MCI’s situated uncertainties and effects, juxtaposing data from experts, markets and patients. For experts, MCI persists because of its conceptual conduciveness to early intervention and research enterprises, its concordance with biomarker hypotheses and its creation of pharma consumers. MCI also justifies anti-ageing markets that treat cognitive health as an individual lifestyle pursuit. For patients, MCI can provide relief (it is not dementia) and the potential to take control, but it can also fuel anxieties regarding future dementia and place people in a liminal zone, neither healthy nor ill. This discussion encourages reflection on the ethical practicalities of diagnoses from different perspectives and brings MCI into dialogue with new dementia and the health politics of ageing.

The third section opens with Bell contextualising new dementia in the history of chronic disease prevention. 20^th^ century public health focused on population-level chronic disease, reconceptualising ‘chronic’ as ‘disabling’. These epidemiologies of chronicity undermined the distinction of ageing and pathology, and posited the longitudinal multi-factor causality of disease, laying prevention’s conceptual foundations. 1970s health promotion made individual lifestyle factors a core preventive concern, while health economics offered welfare-based justifications. Echoing cancer, dementia prevention engaged lifestyle as a medical intervention. Health promotion became cutting-edge biomedical science, with the rational individual being a tool of that science.

Schweda and Pfaller’s chapter notes that new dementia, with its preventive focus, is aligned with successful ageing and anti-ageing notions of ‘good’ personal action today begetting ‘good’ future existence. They argue that common critiques of responsibilisation rarely analyse its moral implications. For instance, the individualisation of responsibility is often cast as inherently bad, but certain political perspectives contend that welfare systems are overextended, and hence responsibilisation addresses an ethical and economic problem. While the chapter is perhaps naïvely reliant on taking scientific knowledge claims for granted, it deftly challenges critical scholarships that forward normative arguments without analysing their moral assumptions.

Foth presents a genealogy of ‘lifestyle’ in preventive public health. Echoing Leanza, he argues that lifestyle-centred neoliberal health regimes have not diminished state involvement. Institutions are central to the coherent manifestation of epidemiology, marketing, research and legislation that (dis)incentivise forms of individual action. Hence, while dementia prevention propels personal responsibilisation, the realisation of that trajectory requires significant institutional effort. While useful, the chapter risks assuming the coherent nature of neoliberalism and couching mundane observation in a terminology of sociological revelation.

Whitehouse and George conclude with an etymological reflection on ‘prevention’ and ‘cure’. Cure seeks to return us to a previous state. When applied to long-term age-related degeneration, it is not clear to what point we wish to be returned, and so curative medicine’s diagnosis dependency requires new pre-disease diagnoses ever earlier in the lifecourse. Prevention is far broader, encompassing various disease stages, stakeholders and contexts. It emphasises the importance of ecologies, providing scope for moving beyond individualist imaginings of cure. The authors conclude that, in foregrounding the lifecourse, new dementia warrants broad reflection on what kinds of society are conducive to good later lives.

I was excited to read this book and it met my expectations. Each chapter offers substantive insight, collectively building a coherent and compelling comment on the present and possible future of new dementia. The authors move beyond classic critical denouncements of neoliberal public health and scientific uncertainty, instead emphasising the complexities of enmeshed individual, professional, state and market considerations. The result is a pleasingly consistent collection of critical takes on critical takes, creating a space for optimism and/or pessimism depending on the reader. I would recommend this book to colleagues and students (indeed, I already have).

There are, however, aspects that could be reconsidered. Claims of an epistemic transition could be tempered. While admittedly weakened in some respects, the neuroreductionist approach remains hugely influential. Aducanumab (incidentally dismissed in chapter two and briefly mentioned in the afterword) is progressing through a high-profile (albeit suspect and likely to fail) bid for Food and Drug Administration approval as the first disease-modifying AD treatment. Moreover, a range of other protein-focused pharmaceuticals are under development, and narrowly neurogenic narratives remain popular in fundraising initiatives. Neuroreductionism remains prominent and is likely more synergetic with prevention than contrasting.

Similarly, MCI is presented as having lost its status as an AD-harbinger, yet mainstream consensus statements continue to champion this potential, even arguing for biomarker-led sub-typification. As such, the extent of the epistemic transformation purported in places could be considered premature, albeit perhaps prescient. Relatedly, some chapters could benefit from Schicktanz’s observation of new dementia’s situatedness. Government, media and epidemiological endeavours are too frequently conflated, when, in practice, issues such as responsibilisation are variably mediated through different stakeholders.

There are noteworthy overlaps with the substantial citizenship literature in dementia studies. It is, therefore, surprising that there is no mention of that critical scholarship. If new dementia is an early life and midlife phenomenon, developing undetected for decades, then we all might be unknowingly living with dementia. In this scenario, sociopolitical arguments about citizenship, disability rights and public effects take on a new character. Yet, despite recurrent discussions of neoliberal governance, and the relations between the ageing person and the state, citizenship is only mentioned once, fleetingly, and without any reference to its existing applications in dementia scholarship.

While points for consideration, these are minor critiques. However, a more substantive issue is the predominance of perspectives from the global north. It is notable that so much critical work on dementia emanates from North America and Europe and that trend is continued here (with the exception of Leibing’s chapter). In fairness, much of the promissory technoscientific story of dementia has been driven by North American and European stakeholders, but with the emergence of ageing and dementia as major global health concerns, these discussions should no longer be so geoculturally constrained.

